# Blood Management Strategies in Placenta Accreta Spectrum

**DOI:** 10.7759/cureus.117351

**Published:** 2026-10-02

**Authors:** Umniyah Abu-Nayla, Atif Bashir Fazari, Kauthar Sulaiman, Asma Fahad, Fareeda Nikhat, Razan Nasir

**Affiliations:** 1 Obstetrics and Gynaecology Residency Program, Dubai Academic Health Corporation, Dubai, ARE; 2 Obstetrics, Dubai Academic Health Corporation, Dubai, ARE; 3 Obstetrics and Gynaecology, Latifa Hospital, Dubai, ARE; 4 Obstetrics and Gynaecology, Dubai Academic Health Corporation, Dubai, ARE; 5 Obstetrics and Gynaecology, Dubai Health Authority, Dubai, ARE

**Keywords:** blood loss management, intraoperative cell salvage, massive transfusion protocol, multidisciplinary team (mdt), obstetric hemorrhage, patient blood management, placenta accreta syndrome

## Abstract

Placenta accreta spectrum (PAS) presents a major challenge in obstetric care because attempted placental separation may result in rapid and life-threatening hemorrhage. As PAS is often suspected before delivery, there is an opportunity to identify correctable risks and prepare for anticipated blood loss. This narrative review examines blood-management strategies throughout the antenatal, intraoperative, and postoperative periods. A targeted narrative search of PubMed and PubMed Central, supplemented by searches of journal and publisher websites, was conducted for literature available through September 2026. Antenatal management focuses on identifying and treating maternal anemia, referring patients to experienced centers, and developing a multidisciplinary delivery plan. Perioperative preparation includes coordination with the transfusion service, timely access to appropriate blood components, clear criteria for activating a major obstetric hemorrhage protocol, and defined responsibilities for each team member. Blood-product preparation should be individualized according to the severity suggested by prenatal imaging and the anticipated anatomical extent of placental invasion, ranging from suspected accreta to increta or percreta. Key intraoperative blood-conservation measures include intraoperative cell salvage and timely administration of tranexamic acid when clinically indicated, particularly during significant hemorrhage. Viscoelastic hemostatic testing, fibrinogen-directed therapy, and selected interventional radiology procedures may also contribute to individualized management. However, evidence supporting these interventions remains inconsistent and is frequently derived from observational studies, expert consensus, or broader obstetric hemorrhage populations rather than PAS-specific randomized trials. Consequently, blood-management plans should reflect the anticipated severity of PAS, the patient’s clinical condition, and the expertise and resources available at the treating institution. A structured and individualized approach linking antenatal optimization, multidisciplinary preparation, timely hemorrhage control, and postoperative surveillance may support safer and more efficient care for patients with PAS.

## Introduction and background

Placenta accreta spectrum (PAS) refers to abnormal placental adherence to or invasion into the uterine wall and includes placenta accreta, increta, and percreta [1]. PAS commonly develops at an area of defective decidualization, often associated with a previous uterine scar, allowing abnormal placental attachment or invasion. Attempted placental separation can disrupt the highly vascular placental bed and cause severe hemorrhage. Its clinical importance is increasing alongside rising cesarean-delivery rates, while placenta previa and previous cesarean delivery are major risk factors. PAS is associated with massive transfusion, coagulopathy, hysterectomy, injury to adjacent pelvic organs, and intensive care admission. For many patients, the standard surgical approach is planned cesarean hysterectomy, with the placenta left in situ to avoid hemorrhage from attempted separation. Conservative or uterus-preserving approaches may be considered in carefully selected patients following thorough counseling at specialized centers [1].

An important feature of prenatally suspected PAS is that the risk of major hemorrhage can often be recognized before delivery, creating an opportunity to plan delivery and prepare for possible transfusion [1,2]. However, available resources and blood-management practices vary across institutions [3,4]. These differences may relate to local expertise, transfusion capacity, and access to cell salvage, viscoelastic testing, interventional radiology, and critical care services [2,4,5]. Complications may result not only from the extent of placental invasion but also from untreated maternal anemia, delayed referral, inadequate blood-component preparation, unclear team responsibilities, and the absence of a coordinated major obstetric hemorrhage pathway [2,3].

Patient blood management is a multidisciplinary approach aimed at optimizing hematologic reserve, reducing avoidable blood loss, and ensuring timely transfusion when clinically required [2]. In PAS, this process begins antenatally with the identification and treatment of maternal anemia [2]. It continues through multidisciplinary operative planning, coordination with the transfusion service, and intraoperative blood-conservation measures [2,4], together with prompt recognition and correction of coagulopathy [3]. The aim is not to avoid necessary transfusion but to reduce preventable blood loss, unnecessary exposure to allogeneic blood products, and delays in treatment [2,3].

Resource mobilization should be tailored to the severity suggested by prenatal imaging and the anticipated extent of placental invasion [1,2,4]. Although definitive FIGO (International Federation of Gynaecology and Obstetrics) clinical grading is determined intraoperatively, prenatal imaging may identify features suggestive of extensive disease or involvement of adjacent organs [1,2]. Suspected disease corresponding to accreta or FIGO grade 1 may require cell-salvage availability and appropriate transfusion preparedness. In contrast, suspected percreta or disease corresponding to FIGO grade 3, particularly grade 3b with bladder involvement, may require greater blood-product availability, experienced pelvic and urological surgical support, critical care capacity, and multidisciplinary assessment of whether interventional radiology would be appropriate [1,2,4,5]. Nevertheless, hemorrhage remains difficult to predict, and adequate preparation is required across the PAS spectrum.

Several strategies have been proposed to improve blood management in PAS, including intraoperative cell salvage, viscoelastic hemostatic testing, fibrinogen-directed therapy, tranexamic acid, prophylactic arterial balloon occlusion, and arterial embolization [2,3,5]. However, the quality of evidence supporting these interventions varies, with many recommendations derived from observational studies, expert consensus, or broader obstetric hemorrhage populations rather than PAS-specific randomized trials [3,5]. Consequently, routine application of every available intervention to all patients may not be appropriate [2,5].

This narrative review addresses this gap by integrating evidence and recommendations across the antenatal, intraoperative, and postoperative periods. It examines hematologic optimization, multidisciplinary planning, transfusion-service preparation, major hemorrhage protocols, goal-directed hemostatic treatment, cell salvage, antifibrinolytic therapy, and selected interventional radiology procedures. Its specific contribution is to distinguish established practices from interventions with uncertain benefit and to present an individualized framework linking anticipated PAS severity with appropriate blood-management resources.

## Review

Search strategy and selection criteria

A targeted narrative literature search was conducted between August and September 2026. PubMed and PubMed Central were searched, supplemented by searches of journal and publisher websites. Search terms included “placenta accreta spectrum,” “patient blood management,” “maternal anemia,” “massive transfusion,” “cell salvage,” “coagulopathy,” “fibrinogen,” “viscoelastic testing,” “tranexamic acid,” “arterial balloon occlusion,” and “interventional radiology,” used individually and in combination. Titles and abstracts were assessed for relevance to antenatal, intraoperative, and postoperative blood management in PAS, followed by evaluation of the available full texts. No formal language or publication-date restrictions were applied. Original clinical studies were prioritized, while guidelines and review articles were retained when required to describe established recommendations or areas in which PAS-specific primary evidence was limited. Reference lists of relevant publications were also examined to identify additional studies.

Eligible publications included human clinical studies involving PAS or broader obstetric hemorrhage when their findings were directly relevant to PAS blood management. Guidelines and reviews were included to describe established recommendations or topics for which PAS-specific primary evidence was limited. Evidence was synthesized thematically across antenatal, intraoperative, and postoperative care. No formal risk-of-bias tool was applied; instead, study limitations were considered according to design, sample size, risk of confounding, and generalizability. No quantitative synthesis or meta-analysis was undertaken because of heterogeneity in study designs, PAS definitions, interventions, and reported outcomes.

Antenatal anemia and hematologic optimization

Antepartum anemia reduces the hematologic reserve available to withstand major blood loss during surgery for PAS [6]. In a multicenter retrospective study of 984 women with PAS, Feng et al. found that antepartum anemia was associated with higher transfusion requirements and an increased risk of adverse maternal outcomes. Following propensity-score matching, patients with anemia received a median of four units of red blood cells compared with two units among patients without anemia. Anemia remained independently associated with adverse maternal outcomes after multivariable adjustment [6].

Evidence regarding anemia treatment specifically in PAS remains limited [7]. Nessar et al. retrospectively compared 72 patients with PAS who received preoperative intravenous ferric carboxymaltose with 71 historical controls. Patients receiving intravenous iron had lower intraoperative and postoperative red-cell transfusion rates and lower overall red-cell and fresh-frozen plasma requirements [7]. However, intravenous iron was administered regardless of baseline hemoglobin or ferritin, and the retrospective use of historical controls limits causal interpretation.

Hemoglobin should therefore be assessed early enough to permit investigation and treatment before planned delivery [1,8]. Oral iron is generally used initially for iron-deficiency anemia, while intravenous iron may be considered when oral treatment is not tolerated, produces an inadequate response, or when more rapid correction is required [8]. Current evidence supports including anemia assessment and correction within antenatal PAS planning, although prospective studies are needed to determine the optimal hemoglobin targets and treatment protocols [1,6-8].

Multidisciplinary planning and scheduled delivery

PAS requires coordinated preparation because the timing and severity of hemorrhage can be difficult to predict [9-12]. International guidance recommends management in experienced centers with maternal-fetal medicine, pelvic surgery, obstetric anesthesia, neonatal, critical care, and transfusion support [1,9]. Depending on the anticipated extent of invasion, additional involvement may be required from gynecologic oncology, urology, vascular surgery, and interventional radiology [1,9]. Antenatal planning allows the operative approach and requirements for blood components, cell salvage, vascular access, and postoperative care to be anticipated [9,10].

Preoperative anesthetic planning should include individualized selection between neuraxial and general anesthesia, with preparation for conversion to general anesthesia when necessary. High-risk cases may require invasive arterial monitoring, large-bore upper-extremity venous access, and rapid-infusion and blood-warming equipment [9]. For stable, asymptomatic patients with suspected PAS, ACOG and SMFM recommend scheduled delivery between 34+0 and 35+6 weeks of gestation, although bleeding, labor, rupture of membranes, pre-eclampsia, or maternal or fetal deterioration may necessitate earlier delivery [1].

Observational evidence suggests that structured multidisciplinary pathways may improve hemorrhage-related outcomes. Luder et al. compared 108 patients managed after implementation of a multidisciplinary protocol with 309 patients treated before its introduction [10]. After adjustment for PAS grade and other clinical factors, multidisciplinary care was associated with lower odds of transfusion, an estimated 260-mL reduction in blood loss, and fewer surgical and urological complications [10].

Antenatal recognition may also facilitate planned multidisciplinary care. In a retrospective study of 57 patients, Jarraya et al. reported lower estimated blood loss among patients diagnosed antenatally than among those with unexpected PAS (1,610 ± 908 mL versus 2,480 ± 1,317 mL), together with lower transfusion requirements [11]. Thang et al. compared 160 planned deliveries with 95 emergency deliveries. Although the proportion receiving transfusion was similar, emergency delivery was associated with a greater mean red-cell requirement (5.3 versus 4.5 units), while planned delivery was associated with later gestational age and improved neonatal outcomes [12].

Overall, available evidence supports antenatal identification, referral to an experienced center, multidisciplinary preparation, and scheduled delivery [9-12]. However, the supporting studies were observational and may have been influenced by disease severity, institutional expertise, and changes in clinical practice over time.

Blood-bank and transfusion planning

The volume of hemorrhage in PAS varies considerably, making individualized transfusion planning essential. The quantity and type of blood components reserved should reflect the anticipated severity of placental invasion, operative strategy, baseline hemoglobin, imaging findings, and likelihood of emergency delivery [13].

Cao et al. evaluated 430 patients with anterior placenta previa, previous cesarean delivery, and suspected PAS, of whom 168 (39.1%) required massive transfusion [13]. Emergency surgery, a higher PAS ultrasound score, lower preoperative hemoglobin, advanced clinical PAS stage, and one-step conservative surgery were independent predictors. A nomogram combining these variables demonstrated strong discrimination in the training and internal validation cohorts, with areas under the curve of 0.908 and 0.925, respectively. Although external validation is required, these findings suggest that combined clinical, hematologic, and imaging assessment may help identify patients requiring greater blood-product availability [13].

Pre-delivery coagulation parameters may provide additional information for hemorrhagic risk assessment [14]. In a retrospective study of 95 patients with invasive PAS, Guo et al. found that higher prothrombin time and fibrin degradation product levels were associated with FIGO grade 3 disease, while elevated D-dimer and fibrin degradation product levels were associated with greater intraoperative blood loss [14]. However, the single-center retrospective design limits the ability to determine whether these parameters can reliably guide prospective transfusion planning.

Considerable variation in transfusion requirements was also demonstrated by Munoz et al. in 142 patients undergoing cesarean hysterectomy for pathology-confirmed PAS. Overall, 85% received at least one unit of blood; 45% received zero to three units, whereas a smaller but clinically important proportion required at least 10 units [15]. These findings illustrate the difficulty of applying a uniform blood-ordering schedule to all patients and support risk-stratified preparation.

Preoperative preparation should include blood typing, antibody screening, cross-matching, and early communication with the transfusion service [16,17]. FIGO guidance recommends that PAS surgery be undertaken where rapid access to a blood bank, massive transfusion protocols, transfusion-medicine expertise, and cell salvage is available [16]. Preparation should include red blood cells and hemostatic components because replacement with red cells alone does not correct dilutional or consumptive coagulopathy [16,17]. During major hemorrhage, plasma, platelets, cryoprecipitate, or fibrinogen concentrate may be required according to the clinical course and laboratory findings [16,17]. Park and Cho similarly emphasize early protocol activation, adequate vascular access, active warming, calcium monitoring, and repeated assessment of hemoglobin, platelet count, fibrinogen, and coagulation parameters [17].

When a major hemorrhage protocol is activated before coagulation results are available, initial balanced transfusion may follow the institutional protocol, commonly approximating a 1:1:1 ratio of red blood cells, plasma, and platelet-equivalent components, before transitioning to laboratory- or viscoelastic-guided therapy [3,16,17]. Ionized calcium should be monitored during rapid transfusion and maintained at approximately 1.1-1.2 mmol/L using intravenous calcium chloride or gluconate as required [17]. The anticipated contribution of cell salvage may be considered when planning cross-matched reserves in selected moderate-risk cases; however, current evidence does not define a safe reduction threshold, and immediate access to additional allogeneic blood must be maintained [16,17].

Overall, PAS blood-bank preparation should combine predefined institutional protocols with individualized risk assessment [13-17]. Massive transfusion packs permit rapid initial resuscitation, but subsequent component therapy should be adjusted to ongoing blood loss and laboratory or point-of-care coagulation results [16,17]. Current evidence does not establish a universally appropriate number of units to reserve for every PAS delivery, supporting an individualized approach based on anticipated hemorrhagic risk [13,15].

Coagulation monitoring and hemostatic management

PAS can cause rapid and massive hemorrhage, requiring continuous assessment of blood loss, hemodynamics, and coagulation [18]. Invasive arterial pressure monitoring and reliable large-bore venous access are often appropriate, while central venous access may be considered when severe bleeding or vasoactive support is anticipated. Close communication among the anesthetic, surgical, hematology, and transfusion teams is essential because hemodynamic instability and coagulopathy can develop rapidly [18].

Conventional coagulation tests, including prothrombin time, activated partial thromboplastin time, platelet count, and fibrinogen concentration, remain important but may be delayed during active hemorrhage [19]. Viscoelastic hemostatic assays, such as rotational thromboelastometry and thromboelastography, provide a more rapid assessment of clot formation and breakdown. These tests may identify fibrinogen deficiency, reduced platelet contribution, coagulation-factor deficiency, and hyperfibrinolysis, thereby supporting targeted administration of cryoprecipitate or fibrinogen concentrate, platelets, plasma, and antifibrinolytic therapy [19].

PAS-specific evidence regarding fibrinogen concentrate remains limited. Akyol et al. retrospectively compared 53 patients who received fibrinogen concentrate with 93 who did not [20]. Patients receiving fibrinogen had greater blood loss, blood product and fluid requirements, and postoperative hospital and intensive care utilization [20]. Because treatment was not randomized and fibrinogen concentrate was likely administered to patients with more severe bleeding, the findings are susceptible to confounding by indication and cannot establish either benefit or harm. They support treatment guided by the clinical course and available coagulation results rather than routine fibrinogen administration to every patient.

A systematic review of 93 studies involving 32,817 participants found that viscoelastic testing could identify hemostatic abnormalities and potentially guide transfusion during postpartum hemorrhage [21]. However, substantial methodological heterogeneity and limited randomized evidence prevented firm conclusions regarding improved maternal outcomes. A randomized pilot trial in postpartum hemorrhage found that ROTEM-guided management did not significantly reduce red-cell transfusion but was associated with reduced plasma administration and a modest reduction in estimated blood loss [22].

Viscoelastic testing may therefore support individualized, goal-directed hemostatic resuscitation in PAS-associated hemorrhage [18,19,21,22]. Nevertheless, results should be interpreted alongside ongoing blood loss, hemodynamic status, and conventional laboratory findings, and testing should not delay emergency transfusion when bleeding is life-threatening [18,19].

Blood-conservation and pharmacological strategies

Intraoperative cell salvage may reduce exposure to allogeneic blood during PAS surgery [23]. In a propensity score-matched study of 102 patients with PAS, cell salvage was associated with reduced dependence on allogeneic blood, with no reported cases of amniotic fluid embolism [23]. However, the retrospective design limits definitive conclusions.

Tranexamic acid inhibits fibrinolysis and may support blood conservation [24]. A multicenter trial involving 1,694 women with placenta previa, including 303 with PAS, found that 1 g of intravenous TXA administered after umbilical-cord clamping modestly reduced postpartum hemorrhage [24]. Because the trial was not limited to PAS, it does not establish routine prophylactic use in every PAS delivery. When TXA is used to treat postpartum hemorrhage, a second 1-g intravenous dose may be administered if bleeding continues after 30 minutes or restarts within 24 hours [1].

Cell-salvage processing includes centrifugation and washing, which substantially reduce soluble amniotic-fluid contaminants [25]. Leukocyte-depletion filters may further reduce residual cellular debris, although they may reduce the rate of reinfusion during rapid hemorrhage [25]. Fetal red cells may remain after processing; therefore, in RhD-negative patients carrying an RhD-positive or unknown-status fetus, the standard anti-D dose of 300 micrograms (1,500 IU) may be insufficient. Fetomaternal hemorrhage should be quantified postoperatively using the Kleihauer-Betke test or flow cytometry to guide additional anti-D dosing [25].

Cell salvage replaces red cells but not platelets or coagulation factors. Hemostatic components may therefore still be required according to the clinical course and laboratory findings [25]. These interventions should complement rather than replace established hemorrhage and massive-transfusion protocols [23-25].

Interventional radiology strategies

Prophylactic arterial balloon occlusion has been proposed as a method of reducing pelvic blood flow and limiting hemorrhage during surgery for PAS [26,27]. However, evidence from original randomized trials has not demonstrated a consistent reduction in transfusion requirements [26,27]. In a randomized trial involving 27 women with suspected placenta accreta, Salim et al. compared preoperative internal iliac artery balloon catheter placement with standard multidisciplinary management [26]. Mean red-cell transfusion requirements and calculated blood loss were not significantly different between the groups, while reversible catheter-related adverse effects occurred in two of the 13 patients who underwent balloon placement [26].

Chen et al. subsequently randomized 100 women with placenta previa and suspected placenta accreta to internal iliac artery balloon occlusion or standard care [27]. Mean packed red-cell transfusion requirements were not significantly different between the balloon and control groups (5.3 versus 4.7 units, respectively; P=0.54). Hospital costs and postoperative fever were higher among patients assigned to balloon occlusion, while other measured outcomes did not differ significantly [27].

Taken together, these trials do not support routine internal iliac artery balloon occlusion for every patient with suspected PAS [26,27]. Decisions regarding its use should consider the anticipated severity and location of placental invasion, the planned surgical approach, local interventional-radiology expertise, and potential vascular or procedural complications. Interpretation of the evidence is limited by differences in PAS definitions and balloon-placement techniques, as well as the relatively small sample sizes of the available trials [26,27].

Postoperative management and future directions

Postoperative care following delivery for PAS should reflect the extent of hemorrhage, surgery, transfusion, and residual placental disease [3,18,28]. Patients with major blood loss or physiological instability may require critical care monitoring, with repeated assessment of hemoglobin, coagulation parameters, fibrinogen, renal function, temperature, and signs of ongoing bleeding [3,18]. When the placenta is intentionally left in situ, surveillance may need to continue after discharge because delayed hemorrhage, infection, coagulopathy, and secondary surgical intervention can occur [28,29].

In their case series and systematic review, Abi Habib et al. identified coagulopathy in 28 of 87 reported cases involving management, with the placenta left in situ. Among the 11 cases with documented timing, coagulopathy developed at a median of 58 days postpartum [29]. Because the evidence was derived largely from selected case reports and case series, this proportion should not be interpreted as the incidence of coagulopathy. Nevertheless, the findings demonstrate that hematologic complications may arise several weeks after delivery and support continued clinical and laboratory surveillance.

Anemia should be reassessed and treated during recovery rather than relying solely on red blood cell transfusion [8,30]. A nationwide analysis of 38,060 women with PAS found substantially higher rates of anemia, postpartum hemorrhage, and transfusion than among women without PAS. Transfused patients also experienced greater peripartum morbidity, although the observational design does not establish that transfusion itself caused these complications [30]. These findings support clinically guided transfusion decisions alongside postoperative iron replacement when appropriate [8,30].

Once hemostasis has been secured, venous thromboembolism risk should be reassessed individually, considering immobility, the extent of surgery, transfusion burden, ongoing bleeding, and coagulation abnormalities [3]. However, PAS-specific evidence defining the optimal timing and duration of pharmacological thromboprophylaxis remains limited [3]. Future research should prioritize prospective multicenter studies evaluating standardized transfusion algorithms, postoperative surveillance, thromboprophylaxis, and patient-centered outcomes. Such evidence may reduce practice variation and clarify which blood-management strategies provide meaningful clinical benefit.

## Conclusions

Placenta accreta spectrum presents a substantial risk of severe obstetric hemorrhage and requires a coordinated blood-management strategy extending from antenatal preparation through postoperative recovery. Early identification and treatment of maternal anemia, planned delivery at an appropriately resourced center, multidisciplinary coordination, and advance communication with the blood bank are central to reducing preventable morbidity. During surgery, rapid recognition of hemorrhage, balanced transfusion, correction of hypofibrinogenemia, and goal-directed coagulation monitoring may help prevent worsening coagulopathy and unnecessary exposure to blood products. Cell salvage and tranexamic acid may provide additional blood-conservation benefits when used in appropriately selected patients and supported by institutional expertise.

Postoperative management should include continued surveillance for anemia, recurrent bleeding, coagulopathy, infection, organ dysfunction, and transfusion-related complications. Patients undergoing placenta-conserving management may require particularly prolonged follow-up because complications can occur after hospital discharge. Thromboprophylaxis should be individualized and initiated only after adequate hemostasis has been established. Although current evidence supports multidisciplinary planning and structured hemorrhage protocols, considerable variation remains in transfusion practices and adjunctive interventions. Prospective multicenter studies are needed to define standardized, evidence-based blood-management pathways and determine which strategies most effectively improve maternal outcomes.
